# Perceived comfort and tool usability during robot-assisted and traditional laparoscopic surgery: a survey study

**DOI:** 10.1007/s11701-023-01785-7

**Published:** 2024-01-13

**Authors:** Jaime Hislop, Oren Tirosh, Mats Isaksson, John McCormick, Chrys Hensman

**Affiliations:** 1 Department of Mechanical Engineering and Product Design Engineering, Swinburne University of Technology, Melbourne, Australia; 2 School of Health and Biomedical Sciences, RMIT, Melbourne, Australia; 3 School of Health Science, Swinburne University of Technology, Melbourne, Australia; 4 Shanghai University of Medicine and Health Sciences, Shanghai, China; 5 Centre for Transformative Media Technologies, Swinburne University of Technology, Melbourne, Australia; 6 Swinburne University of Technology, Melbourne, Australia; 7 Department of Surgery, Monash University, Melbourne, Australia; 8 University of Adelaide, Adelaide, Australia; 9 LapSurgery Australia, Melbourne, Australia

**Keywords:** Robot-Assisted Laparoscopic Surgery (RALS), Traditional Laparoscopic Surgery (TLS), Ergonomics, Injury, Discomfort, Tool usability

## Abstract

It is known that over half of previously surveyed surgeons performing Robot-Assisted Laparoscopic Surgery (RALS) and three-quarters of those performing Traditional Laparoscopic Surgery (TLS) experience intraoperative pain. This survey study aimed to expand upon the ongoing impact of that pain as well as perceived tool usability associated with TLS and RALS, for which considerably less documentation exists. A survey regarding the presence and impact, either immediate or ongoing, of intraoperative pain and Likert scale questions regarding tool usability was administered to TLS and RALS surgeons on the European Association for Endoscopic Surgery (EAES) mailing list. Prevalence statistics as well as trends based on biological sex and glove size were obtained from the 323 responses. Most respondents were right-handed European males (83−88%) with a medium glove size (55.8%). Moderate or severe shoulder symptoms were experienced by one-third of TLS surgeons. Twenty-one percent of RALS surgeons experienced neck symptoms that impacted their concentration. Small-handed surgeons experienced wrist symptoms significantly more frequently than large-handed surgeons, regardless of modality. RALS was associated with a significantly more optimal back and wrist posture compared to TLS. TLS surgeons reported increased ease with applying and moderating force while operating. These results suggest that intraoperative pain may be severe enough in many cases to interfere with surgeon concentration, negatively impacting patient care. Continuing to understand the relationship between tool usability and comfort is crucial in guaranteeing the health and well-being of both surgeons and patients.

**Supplementary Information:**

The online version contains supplementary material available at 10.1007/s11701-023-01785-7.

## Introduction

Studies evaluating the pain prevalence among laparoscopic surgeons are rife in the literature; there are also multiple reviews on the matter [1–3]. However, more nuance is required to draw meaningful conclusions from this data. A previous meta-analysis found that 77.8% of surgeons practicing Traditional Laparoscopic Surgery (TLS) and 53.8% of those practicing Robot-Assisted Laparoscopic Surgery (RALS) experienced pain while operating. In the reviewed studies, the examination of contributing factors primarily focused on demographic or surgical factors, such as biological sex, age, experience, caseload, or operating room ergonomics, rather than tool usability [1]. Numerous studies have found increased risk of discomfort for female and small-handed surgeons, with one study reporting a sevenfold increase in the risk of pain or injury [4]. While this is concerning, further details are required to complete this picture. Most pain prevalence surveys only ask binary response questions, asking whether the surgeon experiences pain in a given region. Comparatively few studies investigate symptom frequency [5–9] or severity [10–12]. Conducting a similar study using Likert scale data and subgroup analysis will highlight how surgical ergonomics are impacted by biological sex and glove size. A Likert scale enables respondents to give a more nuanced, graded response to questions, often using a 1–5 scale from low to high impact. Subgroup analysis breaks down the study sample into subsets of participants in order to better understand if impacts differ across groups with similar traits.

There are limited survey data in the literature making connections between physical comfort, demographic factors, and tool usability. Wong et al. [13] showed that female surgeons were 5−25 times more likely to report the LigaSure device, a pistol-grip operated laparoscopic tool for sealing blood vessels, as being painful or too large to use compared to their male colleagues. The statistical significance of this trend disappeared after adjustment for glove size and other factors, demonstrating that this pattern was in large part due to the smaller female hand size [13]. Sutton et al. found that small-handed females experienced significantly more shoulder and neck discomfort than small-handed males [14]. Previous studies have shown that female surgeons consider TLS tools too large and awkward to use more frequently than their male colleagues [13, 15], as do those with a glove size of 6.5 or below [16, 17]. This often prompts them to adopt a modified one-handed or two-handed grip style [13, 15]. The exact adaptations used for the modified one-handed grip were not captured in the studies and may have been different per impacted user. However, a clear correlation was reported between gender and an inability to use laparoscopic tools in the manner as designed [15]. Minimal information is available regarding the fit and ease of use of RALS hand controls, although Chiu et al. [18] showed that female trainees performed better on simulated suturing tasks using the da Vinci console than their male counterparts.

The excessive force required to operate TLS tools is well documented. Kasai et al. [19] demonstrated that 250 N was required to properly fire an anastomotic stapler. This level of force is sometimes unattainable for female surgeons due to their strength and grip diameter. Poor force transferral means that only 20% of the surgeon’s grip strength translates to the tool tip. The opposite phenomenon is experienced with RALS consoles. The lightweight controls require less applied force to grip and manipulate tissue. Johnson et al. [20] demonstrated that a da Vinci robotic grasper is closed when the hand controls are separated by 4.5°. Full hand control closure may only require approximately 5 Pound per Square Inch (PSI) from the surgeon while the grasper may be applying approximately 500 PSI to the tissue. Mucksavage et al. [21] showed that minor fluctuations in grip forces of less than 1 N may be attributed to the surgeon’s wrist position and prior wear on the robotic tools being used. Increased force variations may be caused by the console model and instrument type. Grip forces ranged from 2.26 to 39.92 N between tools and consoles. The physical separation and utter lack of haptic feedback mean that inexperienced surgeons may not perceive this.

Size, shape, required grip or positioning, and operating force will all have a bearing on the range of surgeons who can comfortably use particular instruments. This survey study aimed to examine intraoperative pain and injury alongside tool usability. The degree to which surgeon discomfort impacted the operation was examined as well as how tool design affected posture and performance during a procedure. Correlations were then explored based on the biological sex and glove size of participants. Evaluating how the experience of using commonly available tools and robotic handle controls differ with anthropometry will provide valuable insights into future surgical instrument design.

## Methods

The survey contained questions on the presence and impact of intraoperative pain or stress, as well as the intuitiveness and comfort of surgical tools for TLS and RALS. Rather than the binary answers elicited in previous studies, responses here were based on how much the level of discomfort interfered with the procedure being performed. To consider tool usability, information was also collected about the pain experienced in 13 regions of the hand. Two questions from the Subjective Workload Assessment Technique (SWAT) [22] were incorporated to evaluate mental effort and stress. Additional questions regarding the ease of use of laparoscopic tools and robotic hand controls were also included. Table 1 contains a summary of the questions and answer formats.

**Table 1:** Survey questions

Question summary	Answer format	Total responses [TLS/RALS]
Information statement	−	−
To confirm your eligibility for this survey: do you have experience in either Traditional Laparoscopic Surgery (TLS) and/or Robot-Assisted Laparoscopic Surgery (RALS)?	Multiple Choice (MC)	323
In which country do you currently reside?	MC	323
Biological sex	MC	323
Age	Text-entry	323
Height	Text-entry	323
Glove size	Text-entry	309
Dominant hand	MC	322
What is your surgical specialty?	MC	323
Years of experience in [TLS/RALS]	MC	[323/102]
On average, how many hours per week do you perform [TLS/RALS]?	MC	[323/102]
What robotic console/s do you use regularly?	MC (multiple answers allowed)	97
Have you at any time in the last 12 months had trouble (such as ache, pain, discomfort, numbness) while performing [TLS/RALS] in any of these regions? If so, what was the severity or impact of the symptoms?	Matrix table of symptom severity in the neck, shoulders, upper back, lower back, elbows, wrists/ hands, hips/ thighs, knees, ankles/ feet	[323/102]
Have you at any time in the last 12 months had trouble (such as ache, pain, discomfort, numbness) while performing [TLS/RALS] in any of these regions? If so, what was the severity or impact of the symptoms?	Matrix table of symptom severity in the thenar, palm, and hypothenar areas, as well as the proximal/middle and distal joints of each finger	[323/102]
With respect to [TLS/RALS]:	Matrix table for level of agreement with prompts regarding positioning, intuitiveness, force, tool fit, and strain while operating	[320/98]
Select which of the following statements generally describes your mental effort during [TLS/RALS]	MC	[318/98]
Select which of the following statements generally describes your psychological stress during [TLS/RALS]	MC	[312/96]
Do you have any additional comments on your experience of performing [TLS/RALS]?	Text-entry	[89/18]
Please indicate if any of the following apply because of the pain you have experienced while operating:	MC (multiple answers allowed)	200
Has the pain you have experienced during TLS and/or RALS (if any) made you consider ending your surgical career?	MC with text-entry	292
Do you have any suggestions on how to improve the ergonomics of TLS or RALS?	Text-entry	90

Similar questions were asked regarding Traditional Laparoscopic Surgery (TLS) and Robot-Assisted Laparoscopic Surgery (RALS). The questions that were asked separately of TLS and RALS surgeons (rather than those posed to all surgeons) are denoted by [TLS/RALS]

A link to the Qualtrics survey was sent to all 8110 email addresses on the European Association for Endoscopic Surgery (EAES) mailing list. The survey was available for completion over a period of 6 weeks throughout July and August 2022. Reminders were sent every 1−2 weeks throughout this time. This study was approved by the Swinburne University Human Research Ethics Committee. An information statement was included in the survey front matter stating the investigator names and affiliations, funding source, question scope, and data handling. A declaration was also included that by commencing the survey, respondents were consenting to participation.

Participants were required to have experience in either TLS or RALS to have their responses considered in the survey. Valid responses were those answering at least one of the questions on intraoperative pain or tool usability. Completion of the demographic questions only was not acceptable for inclusion. Some responses required manual review, as entries were recorded after one week without additional participant input. The number of respondents to each section of the survey was considered during statistical analysis.

Microsoft Excel (Microsoft Corporation, Redmond, WA, USA) was used to obtain basic prevalence estimates for each question. Correlations within the data were determined in the RStudio statistical computing program (RStudio, Inc., Boston, MA, USA). Trends in the results based on biological sex or glove size were of particular interest. Fisher’s Exact Test [23] was used to find patterns in categorical data and determine whether a statistically significant association exists. It allows the testing of significance of categories against groups of participants. A p-value below 0.05 was considered, and referred to, as statistically significant.

## Results

There were 323 valid responses from TLS surgeons collected over the six-week period; 102 respondents also had RALS experience. The most frequently used console for RALS was the da Vinci Xi (77.2%). Most commonly, participants were 47 ± 10.6-year-old males (83%) from Europe (84%), specifically Italy (22% of all respondents), who were 176 ± 8.3 cm tall, of medium (7 − 7.5) glove size (55.8%), and right-handed (87.6%). Female participants were, on average, 7.5 years younger and 12.1 cm shorter than their male colleagues, with significantly smaller glove size. Regarding experience, female participants had significantly fewer years’ experience in TLS (*p* < 0.0005), although not RALS (*p* > 0.05); there was no difference in weekly operating time based on biological sex for either modality. Respondent demographics are included in Tables 2 and 3. The numbers of responses for a given section did fluctuate throughout the questionnaire due to experience (surgeons without RALS experience did not answer these questions), survey dropout, and participants not providing a relevant answer to open-ended or optional questions. The number of respondents for each section is provided in Table 1 alongside the question summary. The total responses for each question were taken into consideration during statistical analysis.

**Table 2:** Responder demographics

Biological sex	Female	Male
Biological sex (*n*)	55	268
Age (mean ± standard deviation)	40.7 ± 9.4	48.2 ± 10.4
Height (cm) (mean ± standard deviation)	166.9 ± 5.0	179.0 ± 7.3
Glove size		
Small (≤ 6.5 or S)	44	8
Medium (7 − 7.5, or M)	10	171
Large (≥ 8 or L)	0	76
Dominant hand		
Right	282	
Left	6	
Ambidextrous	34	
Continent		
Africa	6	
Antarctica	0	
Asia	40	
Australia/Oceania	0	
Europe	270	
North America	5	
South America	2	
Specialty		
Bariatric	24	
Colorectal	75	
Gastrointestinal	56	
General	138	
Gynecology	2	
Urology	1	
Other	27

**Table 3:** Surgeon experience

Surgery modality		TLS (*n* = 323)		RALS (*n* = 102)	
		Female	Male	Female	Male
Years’ experience	0 to 2	6	2	15	44
	3 to 5	10	21	6	19
	5 to 10	14	50	0	11
	10 to 15	14	62	0	3
	15 +	11	133	1	3
Hours operating per week	0 to 1	0	3	12	27
	1 to 5	16	47	6	32
	5 to 10	24	105	3	13
	10 to 15	9	58	1	7
	15 to 20	5	35	0	1
	20 +	1	20	0	0

**Fig. 1:**
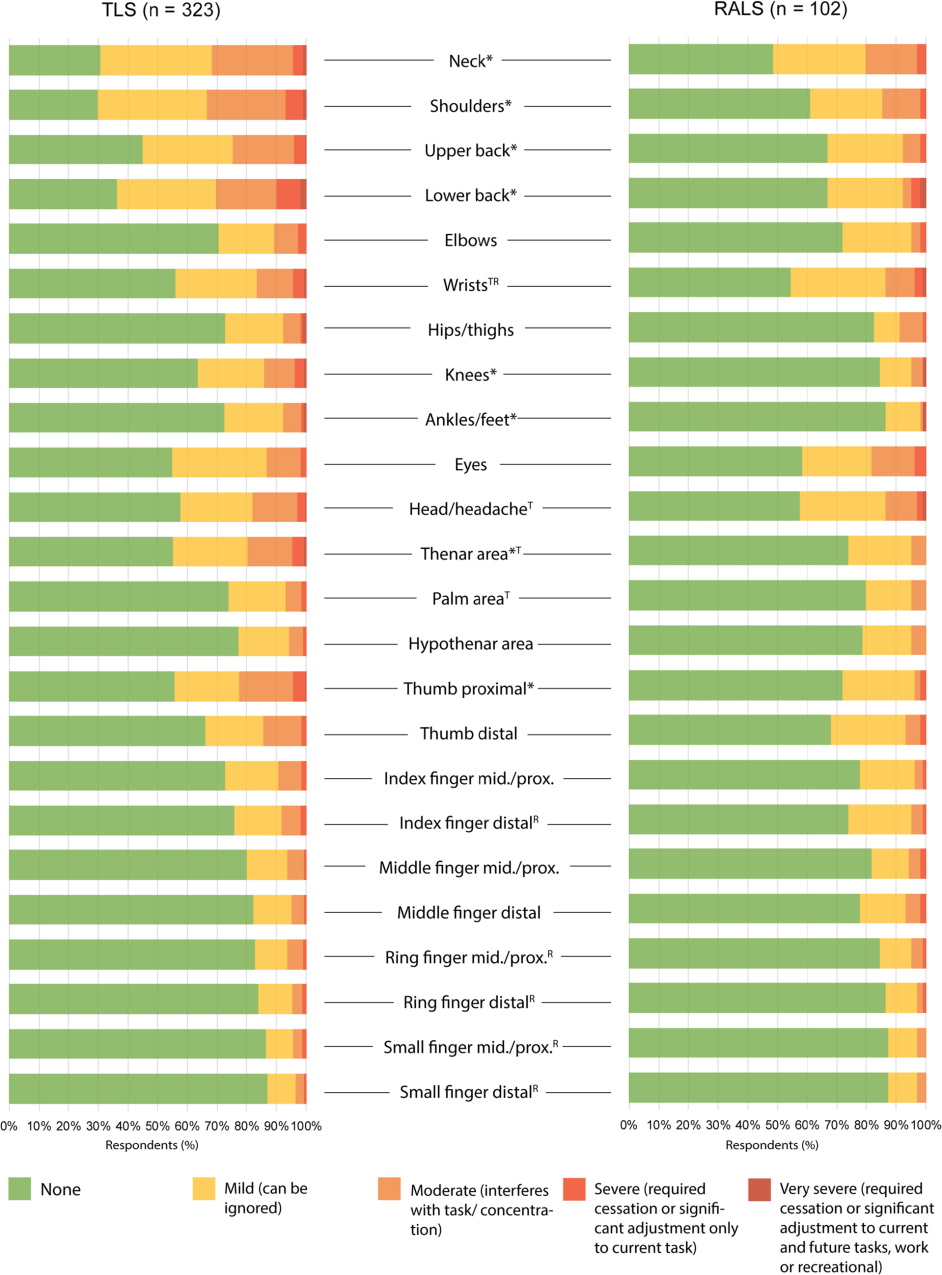
The severity (determined by impact) of intraoperative pain experienced by surgeons during Traditional Laparoscopic Surgery (TLS) and Robot-Assisted Laparoscopic Surgery (RALS). The asterisk *represents a significant difference (*p* < 0.05) between the responses given by TLS and RALS surgeons for the part of the body in question. The T symbol represents significant differences when responses were stratified by biological sex or glove size for TLS and the R symbol correspondingly for RALS

The shoulder and neck were the sites of the most complaints reported by TLS (70%) and RALS surgeons (39−52%); these were also the locations of the largest proportions of moderate and severe pain. TLS surgeons experienced a significantly increased severity and impact of pain compared to RALS surgeons for the neck, shoulders, upper and lower back, thenar area, proximal phalanx of the thumb, knees, and ankles and feet. Figure 1 shows the severity of pain reported by TLS and RALS surgeons.

Operating laparoscopically required moderate mental effort for 56−57% of surgeons regardless of modality. However, a significantly larger proportion of surgeons reported that the robotic console caused moderate to high stress and confusion compared to TLS (49.0 vs 34.9%, *p* < 0.03).

**Fig. 2:**
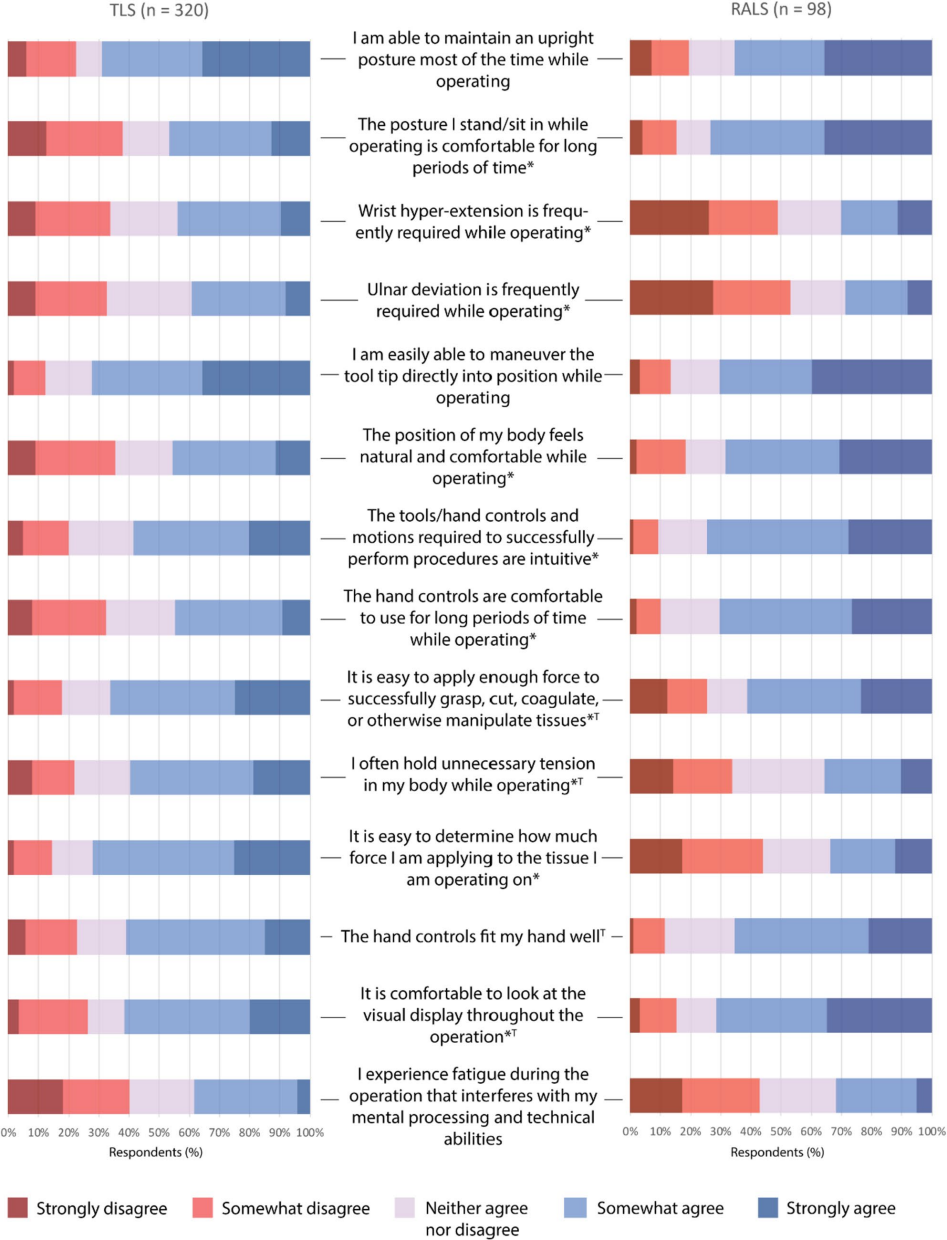
The usability of tools reported on a Likert scale by Traditional Laparoscopic Surgery (TLS) and Robot-Assisted Laparoscopic Surgery (RALS) surgeons based on the comfort and intuition of tools and the position adopted while using them. The asterisk * represents a significant difference (*p* < 0.05) between the responses given by TLS and RALS surgeons. The T symbols represent significant differences when responses were stratified by biological sex or glove size for TLS. There were no differences for RALS based on biological sex or glove size

Overall, surgeons agreed on the usability of RALS equipment with significantly more consistency than TLS. This information is depicted in Fig. 2. Most RALS surgeons agreed or strongly agreed on the perceived naturalness and comfort of their operating positions (68%); TLS surgeons had mixed reports, with 35% disagreeing on their intraoperative comfort, and 45% agreeing. The difference in the dispersion of answers between TLS and RALS surgeons was statistically significant (*p* < 0.05). Surgeons experienced significantly more comfort viewing the RALS console display than a TLS monitor. Additionally, surgeons were significantly more likely to hold unnecessary tension while performing TLS compared to RALS. RALS controls were associated with intuitiveness and comfort at a significantly higher frequency than TLS tools. Surgeons were more likely to confirm that TLS required regular wrist hyper-extension and ulnar deviation compared to RALS (*p* < 0.05). RALS surgeons found it more difficult than TLS surgeons to determine the level of force they were applying to tissue (*p* < 0.05), although 34% still reported that this was easy to do.

Similar trends were found for surgeons that were female and those that were small-handed, as most female surgeons had a glove size of 6 or 6.5. Female and small-handed TLS surgeons reported increased headaches, as well as pain in the wrists and thenar area compared to their male colleagues. Those with a glove size of 6 or 6.5 experienced significantly more palm pain than larger-handed surgeons (*p* < 0.05); this difference was not significant when stratified by biological sex. Female and small-handed surgeons were less likely to report that TLS tools fit their hands well compared to their colleagues (*p* < 0.05). Female surgeons were twice as likely to find it uncomfortable looking at a TLS monitor throughout an operation (38.9 vs 19.6, *p* < 0.05). Male surgeons were significantly more likely to report mild pain in the ring and little fingers during RALS than female surgeons.

Two-hundred surgeons reported requiring interventions to investigate or alleviate the pain. Of these 200 participants 71% used pain medication, 39.5% were engaging in physiotherapy, 10−12.5% were taking leave, visiting a doctor, or receiving medical scans, and 4% required surgery. Intraoperative pain made 8.9% of surgeons consider ending their surgical career. Female surgeons reported utilizing these interventions and considering retirement at slightly higher frequencies than their male colleagues.

## Discussion

Compared to a previously conducted meta-analysis [1], the prevalence rate of neck and shoulder symptoms of any severity was approximately 20% higher for both TLS and RALS. Several previous survey studies provide data on the perceived severity of intraoperative pain during TLS on a scale given in either a numerical or descriptive (i.e., from mild to severe) terms for different anatomical sites [10–12]. Tjiam et al. [10] found that the majority of surgeons experiencing symptoms considered them to be mild, which is consistent with the present study, whereas Wauben et al. [11] and Wells et al. [12] found that a notable proportion was experiencing moderate symptoms in various anatomic regions. The shortcoming of the rating scales used in these previous studies is highly subjective and lack context. This was addressed in the current study by referring to the pain in terms of how it impacted current and future tasks. The only data found in the literature on the severity of pain during RALS were an overall score presented by Wells et al. [12]. Therefore, the results presented here stratified by anatomic region provide important insight. The wrists, ring fingers, and small fingers were sites of significant differences in the pain reported by female and male RALS surgeons, suggesting that all RALS surgeons may benefit from refining the design of existing robotic controls.

Previous studies have surveyed surgeons regarding the perceived usability of particular TLS tools to investigate equipment design. These studies showed that female and small-handed surgeons find most tools more difficult to use than their male colleagues, and often must adapt their grip accordingly [13, 15]. Results were expressed in terms of awkwardness and fit, which provide limited insight on how instrument design impacts operative success. The present study posed questions on more practical concepts including positioning, intuitiveness, force, tool fit, and fatigue. These questions were intended to have clear implications for the ease of operating using TLS and RALS. Additionally, the prompts regarding posture, force, and fatigue were all indicators for ergonomic risk factors. Points of concern for TLS centered around surgeons adopting unhealthy back and wrist positions throughout procedures. There is limited data on perceived tool usability based on biological sex for RALS in the literature. No significant differences were found between the results provided by male and female surgeons regarding console usability in the results presented here. This perceived ease of performing RALS regardless of biological sex supports the notion that robotics may improve the gender equity in the operating room.

This is a pivotal time to be investigating tool usability in relation to robotic surgery, as several new systems are currently entering the market with different approaches to controller design. Form, intuitive movement, and placement of various function controls all contribute to a system’s ease of use. The Senhance system from Asensus Surgical has scissor-like handles and a mirrored working direction to emulate the experience of performing TLS. In contrast, the manipulators of CMR’s Versius and Medtronic’s Hugo are closer in design to different game controllers and utilize in-line movements [24]. Anthropometry and user feedback regarding the functionality, form, and size of prototypes were considered in the design of the Versius controllers [25]. These three systems all have open or semi-open consoles [24]. Hand control and console design will change the way surgeons are positioned while operating and likely have a notable impact on comfort.

Survey length was a limitation of this survey. It was estimated to take 10−20 min to complete. While efforts were made to streamline the process using survey logic, the length may have been off-putting or prohibitive for some surgeons. This may have contributed to the decline in respondents observed throughout the questionnaire. Additionally, matrix tables may lead to missing data, despite their efficiency from a survey design point of view [26, 27]. Additional limitations included selection and recall bias, which are present in many survey studies.

While the focus of this investigation was the prevalence of physical discomfort and pain related to the use of TLS and RALS tools, studies have raised the effects of mental load on the well-being of surgeons [28]. Non-technical surgical skills including situational awareness, decision-making, stress resilience, communication and leadership requirements add to the mental workload impacting surgeon performance. Future work could investigate both the physical and mental pressures on surgeons to expand the understanding of their workplace challenges.

In conclusion, the results presented here build on the existing picture emerging in the literature that RALS is easier and more comfortable for surgeons to perform than TLS. Up to one-third of TLS surgeons and 21% of RALS surgeons considered their symptoms to be moderate or severe, negatively impacting their operating performance. Methods to support or change wrist and back posture during TLS would greatly benefit surgeons. Opportunities also exist to improve upon the viewing angle and hand controls of RALS consoles to further benefit surgeon comfort. As new robotic systems enter the market, it is expected that their novel design features will positively impact surgical ergonomics. Continuously aiming to understand and improve upon laparoscopic tool design is an important pursuit to support surgeon well-being.

## Supplementary Information

Below is the link to the electronic supplementary material.

Supplementary file1 (XLSX 375 KB)

## Data Availability

All data generated or analyzed during this study are available from the corresponding author on reasonable request or on figshare at 10.6084/m9.figshare.24975273.
